# Connecting knowledge and practice: specialization course in dentistry in public health at Brazilian unified health system - a journey of transformative integration

**DOI:** 10.1186/s12909-025-06987-1

**Published:** 2025-03-21

**Authors:** Afonso Luís Puig Pereira, Danielle de Costa Palacio, Danielle Viana Ribeiro

**Affiliations:** 1 Hospital Israelita Albert Einstein e Universidade Federal de São, São Paulo, Brazil; 2 Hospital Israelita Albert Einstein - São, São Paulo, Brazil

**Keywords:** Teacher-assistance integration services, Internship and residency, Postgraduate education in dentistry, Training of health workforce, Unified health system

## Abstract

**Objective:**

To understand the teaching-learning process from the perspective of professors, students, preceptors, managers, and the community through an internship of a specialization course in dentistry in public health, based on the educational strategy of teaching-service-management-community integration within the Unified Health System (SUS).

**Methodology:**

: A Case Study was carried out with a descriptive and exploratory approach, originating from a pedagogical activity that took place in the territories served by Primary Health Care Services in the city of São Paulo, Brazil. Participants included professors and students from a specialization course in dentistry in public health with training in the SUS, preceptors, managers, and residents from communities where internship activities occurred, thus encompassing all aspects of the teaching-service-management-community integration. The research utilized convenience sampling for participant selection and conducted descriptive analyses of demographic data. Qualitative data were analyzed using thematic content analysis, while quantitative data were processed through descriptive statistical analysis.

**Results:**

There was little ethnic representation: professors were predominantly white men; students and health professionals were mostly white women; and community residents were mostly white or brown men. It was observed that the collaboration between the education institution, students, and healthcare professionals (preceptors) in practical settings, using strategies such as area recognition, teamwork, and home visits, which are planned before the internship in workshops with preceptors, promotes the integration of teaching-service-management-community for training in public health. Although national health training policies favor this integration, sectoral management remains disconnected from the educational process, as does the community.

**Conclusion:**

Social inequalities are mirrored in education, and thus ethnic representation can contribute to bridging this gap. In this experience, the success of the integration of teaching-service-management-community resulted from the pedagogical collaboration between the educational institution and healthcare professionals, with management and the community remaining separated as passive subjects in the educational process.

**Supplementary Information:**

The online version contains supplementary material available at 10.1186/s12909-025-06987-1.

## Introduction

To develop skills in health professionals through the connection between students and the social reality, where the real population needs are found, the teaching-service-management-community integration stands out, known as the Training Quadrilateral [1]. Is an educational strategy where it is possible to learn about and experience health policies in the territories, as well as the work process and practices of teams from all professional categories, training students in and for the Brazilian public health system, called Unified Health System (SUS). This praxis, in the sense of what Paulo Freire referred to as action-reflection, aligns with the constitutional premise of the ordinance on health workforce [2] and simultaneously encourages the transformation of practices through the interaction between professionals, students, and users. This interaction is based on critical reflection on health work to meet population demands [1, 3, 4]. This training framework is implemented in the real work scenario, where professors (teachers), students, health care professionals (preceptors), managers, and the population (community residents) share their experiences [5], fostering collaboration between all parties.

In the Training Quadrilateral [2], Education Institutions entrust their teachers with the responsibility of training students in various areas, including academic-scientific, ethical, cultural, and humanistic domains. Additionally, they are responsible for disseminating the knowledge acquired through services and social movements to promote technical and professional development. The health service, by preparing to welcome students as preceptors, is responsible for inspiring and integrating students into a real-life work scenario, developing the skills and competencies expected from the training of future professionals [6]. Conversely, sectoral management, represented by state managers, ensures compliance with public policies [7] and must provide the necessary resources for the development of teaching, research, and extension, as well as support democratic rights. Complementing the integration, the population, central to the training policy in health, in its constitutional role, must be active participants, not mere spectators, in the teaching-learning process [2]. It is noteworthy that studies indicate that the integration of teaching, service, management, and community fosters critical-reflective training in real practice settings. This approach contributes to the formation of professionals with a deeper understanding of the reality experienced by the population and brings benefits to healthcare services [8–12].

Although successful experiences [3–5] support the integration of teaching-service-management-community and are extensively analyzed in the literature, such as the National Program for the Reorientation of Professional Health Education and the Education Program for Health Work [13, 14], it is still necessary to study it in depth to better understand its convergences and divergences in the training process within the scope of graduate studies. Therefore, an interpretative effort of the educational practices and discourse developed in graduate professional training is warranted, involving those engaged in the construction of this educational process and identifying potentialities and possible weaknesses in training. Thus, the main objective of this study was to understand the teaching-learning process from the perspective of faculty, students, preceptors, managers, and the community through a postgraduate internship in dentistry in public health, based on the educational strategy of teaching-service-management-community integration within the SUS in São Paulo, Brazil.

## Methodology

### Study context

This work stemmed from a pedagogical activity as part of the specialization course in public health dentistry at Hospital Israelita Albert Einstein, which provides both theoretical and practical training through a practical internship. The setting for this activity was the territories served by Primary Health Care Services in the neighborhoods of Campo Limpo and Vila Andrade, located in the southern region of the municipality of São Paulo, Brazil. This was part of a partnership established in 2001 between the Hospital Israelita Albert Einstein and the Municipal Health Department of São Paulo for the management of health services. This region, with a population of 400,198 individuals [15], is characterized by several challenges related to social inequality.

Before receiving the students of the specialization course, dentists from the internship-providing Primary Health Care Services were invited to conduct preceptorship workshops. These workshops were planned by the course coordination and included two four-hour meetings. During these meetings, a comprehensive introduction and in-depth discussion on the topic were structured for service professionals. The aim was to train them, provide context about the students’ situation in the course, and plan internship activities. Important topics discussed in the workshops included concepts, profiles, and skills desired by the preceptor, the teaching-learning process, teamwork, and teaching-service-management-community integration. Additionally, challenges such as evaluation, internship planning, and insecurities regarding the exercise of the profession were extensively discussed. This process facilitated an alignment with the course theory, an understanding of concepts related to preceptorship, teaching-learning strategies, internship planning, and directed study. It also fostered greater integration between the course coordination and the future preceptors.

Two distinct classes, one in 2021 and another in 2022, totaling 30 students, were divided into seven tutorial groups in their respective years, supervised by one to three preceptors depending on the circumstances. Each tutorial group remained at the same Primary Health Care Service throughout the internship. Planning of field immersion activities, developed by the course coordination in collaboration with preceptors, aimed at training public health dentists, included following the routine of the Oral Health Teams (OHT), familiarization with the Primary Health Service and the assigned territory, team meetings, shadowing in management, nursing, medical, and dental consultations, visits to health facilities within the territories such as Mental Health Care Services, Urgences Health Care Services, and Pediatric Health Care services, home visits, collective actions within the territory or in schools, visits to Non-governmental Organizations, and other relevant activities according to the territory visited.

### Participants

The recruitment of research participants was carried out during the internship activities, where participants from all parts of the teaching-service-management-community integration were invited: 27 professors, 31 students, 10 preceptors, and 15 health managers. No responses were obtained from municipal managers. Nine professors, sixteen course students, ten health professionals/preceptors from the internship-providing Primary Health Care Services, and nineteen community residents registered at the internship-providing Primary Health Care Services where activities were carried out agreed to participate in this research. Convenience sampling was used, and data were collected between May and June 2022. The recruitment of research participants was tailored to the specificities of each segment involved. Professors and managers were invited by email, students, and preceptors by email and text message by cell phone, and community residents were invited during the internship activities.

### Inclusion and exclusion criteria

The inclusion and exclusion criteria for study participants are described in Chart 1.

**Chart 1:** Eligibility criteria for research participants

**Segment participating in the research**	**Inclusion criteria**	**Exclusion criteria**
Teacher	Health professional or of a related area part of the faculty of the course on Public Health Dentistry.	Leave/health leave during the study period; Member of the group of researchers of this study; Opting out of the study.
Student	Dental surgeon actively registered in the trade association and student of the course having completed the activities related to the internship.	Leave/health leave during the study period; Opting out of the study.
Preceptor	Health professionals from the internship provider Primary Health Care Services who participated in the preceptorship workshop prior to the internship.	
Manager	Professionals of the Municipal Health Department of the municipality of São Paulo.	
Community resident	Registered in one of the internship-provider Primary Health Care Services. Age ≥ 18. Be legally responsible or have been attended by a course student or preceptor.	Have any clinical condition that makes participation in the research unfeasible; Opting out of the study.

Source: created by the authors

### Data collection

Professors, managers, students, and preceptors who met the inclusion criteria received the Informed Consent Form and a link to a semi-structured questionnaire by email. This questionnaire included open and closed questions distributed across two thematic blocks: one covering the socioeconomic profile of the participants (age, gender, and self-reported skin color) and the other assessing the perception of the teaching-learning process and teaching-service-management-community integration.

For students, a documentary analysis of field diaries was carried out as part of an evaluative activity. Additionally, two questions were asked before and after the internship to assess learning.

For preceptors, a focus group session was conducted, lasting one hour and thirty minutes, with the participation of seven professionals. This session facilitated interaction among participants, allowing for the recording of diverse viewpoints and opinions, and resulted in the joint construction of the meanings of the topics determined by the researchers [16].

Community residents who expressed interest in participating in the study during the health activities carried out by students and preceptors in the territory were invited to sign a printed Informed Consent Form. They then completed a questionnaire that included questions about their sociodemographic profile, their perceptions, the time and quality of care they received, and their knowledge of popular participation.

The development of the individualized semi-structured questionnaire for each segment of the Training Quadrilateral was guided by questions related to the course, highlighting the proposed internship activities and the participants’s perceptions of the teaching-learning process.

### Study design and data analysis

To analyze the teaching-service-management-community integration based on the internships of the classes that had completed the course, a single Case Study [17] was implemented. Descriptive qualitative data were collected through document analysis, the application of semi-structured questionnaires, and the conduction of a focus group. These data were analyzed using thematic content analysis, following the method described by Minayo (2013) [18]. Descriptive statistical analysis was employed for the sociodemographic profile data of the participants. After processing and interpreting the collected data, it was observed that no new relevant dimensions related to the theme emerged, indicating that the data had reached a saturation point. All analyses were carried out under the “Organizational Contract of Public Action for Teaching-Service,” duly contracted with municipal management.

### Trustworthiness and rigor

This research followed the SRQR (Standards for Reporting Qualitative Research) guidelines [19] to ensure comprehensive and transparent reporting of the qualitative methods and findings. Both sets of guidelines emphasize thorough documentation of the study’s methodology, participant recruitment, data collection processes, and analysis to improve the rigor and replicability of qualitative research. Triangulation of data collection sources was conducted to ensure a more comprehensive and reliable analysis, incorporating multiple perspectives and validating findings across different sources.

The authors and researchers of this study are specialists in public health and coordinators of the specialization course in public health dentistry. They are familiar with both the educational institution and the research field. As a result, all respondents were fully aware and agreed before signing the informed consent form for the research. With full awareness and integrity, they chose to investigate the teaching-learning process implemented through teaching-service-management-community integration.

### Research ethics

This study was conducted in accordance with CNS Resolution 466/12 and was approved by opinion No. 5,342,647. The informed consent to participate was obtained from all the participants in the study. To preserve the anonymity of the participants, the letters T (teacher), S (student), P (preceptor), M (manager), and C (community resident) were used to refer to them throughout the text.

## Results

This section presents the key themes and sub-themes that emerged from analyzing the profile and interviews conducted with teachers, students, health professionals, and community residents, exploring their experiences in the teaching-service-management-community integration (Table 1).

**Table 1:** Key main theme and sub-themes

Number	Theme	Sub-theme
Theme 1	Profile of participants	Sociodemographic data
Theme 2	Integration from the perspective of the players: teaching-service connection	a. Continuing education actions b. Recognizing the role of the preceptor c. Internship as a journey of transformative integration d. Perception of the players of common activities e. Synthesis of the day f. Focus areas for enhancement
Theme 3	Integration in disconnection with management and the community	a. Lack of knowledge about the internship b. Passive agent

### Theme 1: profile of participants

With regard to the sociodemographic profile, the majority of teachers (77.8%), students (75%), and health professionals (80%) who responded to the survey identified as white, indicating limited participation from individuals of Black and Brown racial backgrounds. Furthermore, the teaching cohort predominantly comprised males (77.8%), whereas the student body (68.7%) and health professional group (60%) were predominantly female.

The sociodemographic data from this study demonstrate the predominance of white and male professionals in the field of public health, particularly in dentistry, as illustrated in Table 2.

**Table 2:** Sociodemographic characteristics of teachers (*n* = 9), students (*n* = 16), preceptors (*n* = 10), and community residents (*n* = 19) who answered the survey on the internship of the public health dentistry course at hospital Israelita Albert Einstein. São Paulo/SP. 2022

Research Participant	Teachers	Students	Health Professionals	Community Residents
Sociodemographic Data	*n* (%)			
Black Male	0	1 (06.2)	0	0
Brown Male	0	0	0	5 (26.3)
White Male	7 (77.8)	4 (25.0)	4 (40.0)	6 (31.5)
Black Female	1 (11.1)	1 (06.2)	1 (10.0)	0
Brown Female	1 (11.1)	2 (12.5)	0	4 (21.1)
Yellow Female	0	0	1 (10.0)	0
White Female	0	8 (50.0)	4 (40.0)	4 (21.1)
Mean Age (years)	39.4 [28–67]	36.7 [25–64]	41.9 [32–52]	30.1 [4–92]

Source: created by the authors

### Theme 2: Integration from the perspective of the players: teaching-service connection

#### Continuing education actions

The subsequent excerpts demonstrate the positive perception of preceptors regarding the workshops conducted:

The workshops were fundamental to offering a more targeted and grounded preceptorship. I hadn’t had the opportunity to play that role yet. (P4)

Training was fundamental, it provided support and planning […] how to create the schedule, what to do, and how to do it. (P5)

[The workshops] were important to level out and standardize the internship, allowing it to offer similar opportunities and actions. (P7)

The study also highlighted the preceptors’ learning, as they leverage the daily service dynamics to optimize and reorganize workflows.

I got to know the territory more deeply because I visited areas that are not my health responsibility and had contact with many more professionals from the Primary Health Care Services and other health services. A lot of knowledge was exchanged with the students and other professionals I brought to the students. Rich learning will be reflected in my care routine. (P1)

In this practice, we were able to interact and learn more about other areas of our own unit, reaffirming ourselves in the universe of our responsibility and reflecting on our own role. (P6)

It was a way of reflecting on the service (P7).

#### Recognizing the role of the preceptor

Acknowledging the preceptor’s role is essential, as they are fundamental to the teaching-service-management-community integration.

The financial return is also motivating. Sharing knowledge with students while still having the opportunity to be paid was a relevant point. (P4)

Compensation is an important point. (P7)

#### Internship as a journey of transformative integration

The internship field constitutes a significant differentiating factor in the teaching-learning process, as evidenced by the statements of teachers, students, and preceptors:

Having an encompassing view of the functioning of the Primary Health Care Service, not only of the dental service, but also of the service flows of other professionals, such as administrative technicians, nursing technicians, nurses, and physicians. The care in the organization of family records, how the dental records are filled out (I immediately adopted this at my Primary Health Care Service), and the emphasis on patient safety. (S1)

The internship was essential to put into practice what was taught in theory throughout the course. (S2)

The internship was one of the most enriching experiences of my personal and professional life. It allowed me to burst the bubble in which I lived. (S8)

I believe that the course was essential for my knowledge and self-knowledge as well. I certainly completed it with a different perspective, happy to meet people who make me believe in society and in caring for others. (S4)

Students are eager for the internship and still see it as a differential of graduate studies. It is an excellent opportunity for the students to experience the reality of what they learn in theory. (T7)

It is a differentiated internship that provokes in students the opportunity to learn in loco and through reality. It really is what makes a difference in the course. (P1)

Students have a theoretical background. However, the internship brings the opportunity to develop “know-how” skills. (P4)

#### Perception of the players of common activities

**Graph 1:**
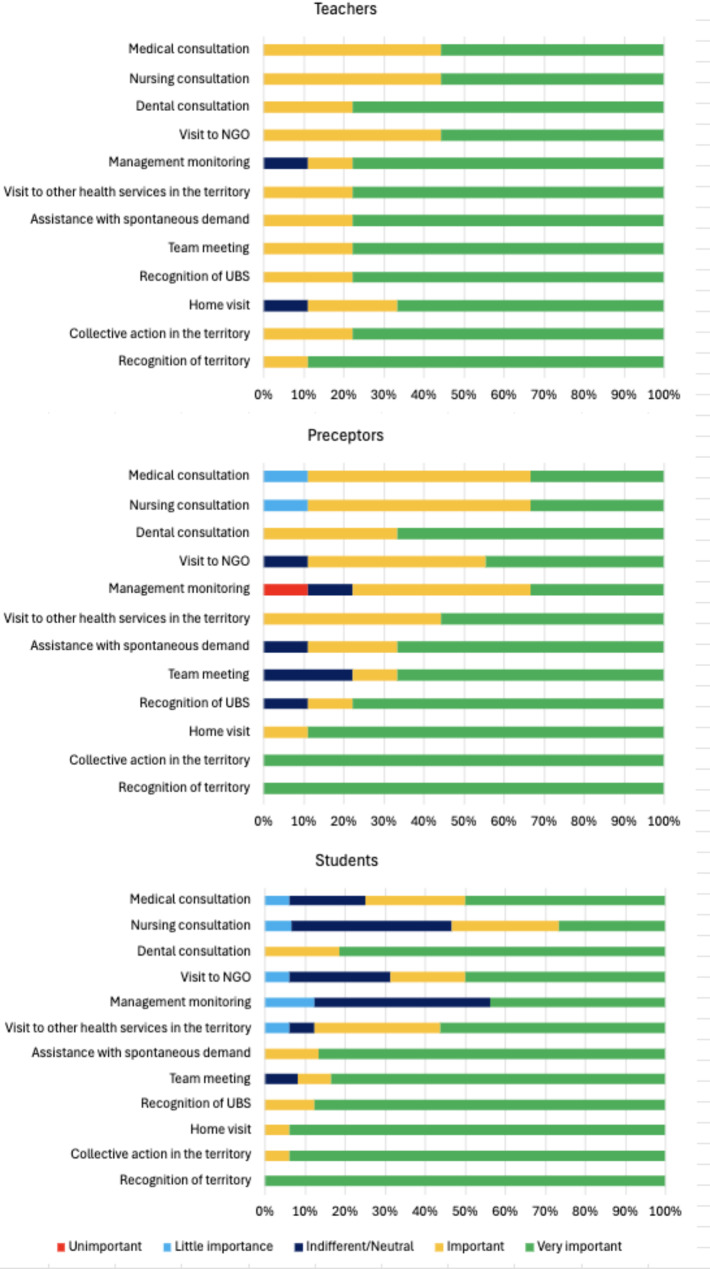
Likert Scale on the Perception of the Importance of the Activities Carried Out with the Students During the Internship According to the Segment Questioned: Teachers (*n* = 9), Preceptors (*n* = 10), and Students (*n* = 16). Internship of the Public Health Dentistry Course at Hospital Israelita Albert Einstein. São Paulo/SP. 2022

Graph 1 illustrates the perceptions of teachers, preceptors, and students regarding twelve common activities, previously agreed upon with the three Primary Health Care Services. These activities are emphasized based on their perceived significance for training in public health, with particular attention to those considered more or less critical. It is noteworthy that students did not provide responses for three items, which accounts for the observed gaps. Among the various possibilities, each Primary Health Care Service could develop pedagogical strategies tailored to its specific territorial context and available human resources during the internship period, going beyond the activities planned by the course coordination and preceptors.

Some utterances by students and preceptors highlight the importance of these strategies:

*Learning about the territory* made me aware of how important it is to know where one operates, and how important it is to have reality ‘’mapped out’’ to plan any action. (S7)

[the *home visit*] made it possible to know the patients and their needs, as well as the vulnerability they are exposed to. (S14)

*Collective action* [in the territory] was the most rewarding aspect, as it required prior knowledge of their territory, creativity, patience, ability to work as a team, and resilience to deal with unexpected situations. (S10)

*Learning about the territory* puts students face to face with the determinants of health; *home visits* expose important perceptions about social determinants in health; and *collective action in the territory* makes them reflect on the possibilities of health education, development of territorial actions, and personal potentialities. (P1)

#### Synthesis of the day

At the conclusion of each day, the students’ observations and experiences were critically analyzed as a pedagogical strategy for consolidating learning. Through a structured group discussion, a synthesis was conducted, which was subsequently emphasized by students and preceptors as a significant component of continuing education:

The synthesis at the end of the day was super important and I would even like the synthesis time to be longer. (S4)

The synthesis at the end of each day is an incredible way to consolidate knowledge for both students and professionals. (P7)

The synthesis was a great learning opportunity because it made us reflect on the whole day, on each action, and on what we could do to improve our daily work. (P10)

#### Focus areas for enhancement

Two significant challenges were identified in this integration process: one pertaining to the limited duration of the internship and the other concerning the inadequate training provided in dental undergraduate curricula.

I believe that I could not take as much advantage of it as I would like because there was too much information in a short time. (S1)

There are many processes to explain and present […] I am sure that some processes were confusing for them. (P3)

We had to constantly reinvent ourselves because we were faced with situations of difficulty in the basics. (P9)

During certain phases of the internship, the fundamental principles of dentistry, which should have been acquired during undergraduate studies, demonstrated inadequate preparation. Based on the identification of topics that necessitated greater comprehension, the theoretical instruction provided by the preceptors aimed to address these knowledge deficiencies at an appropriate juncture.

### Theme 3: Integration in disconnection with management and the community

#### Lack of knowledge about the internship

**Graph 2:**
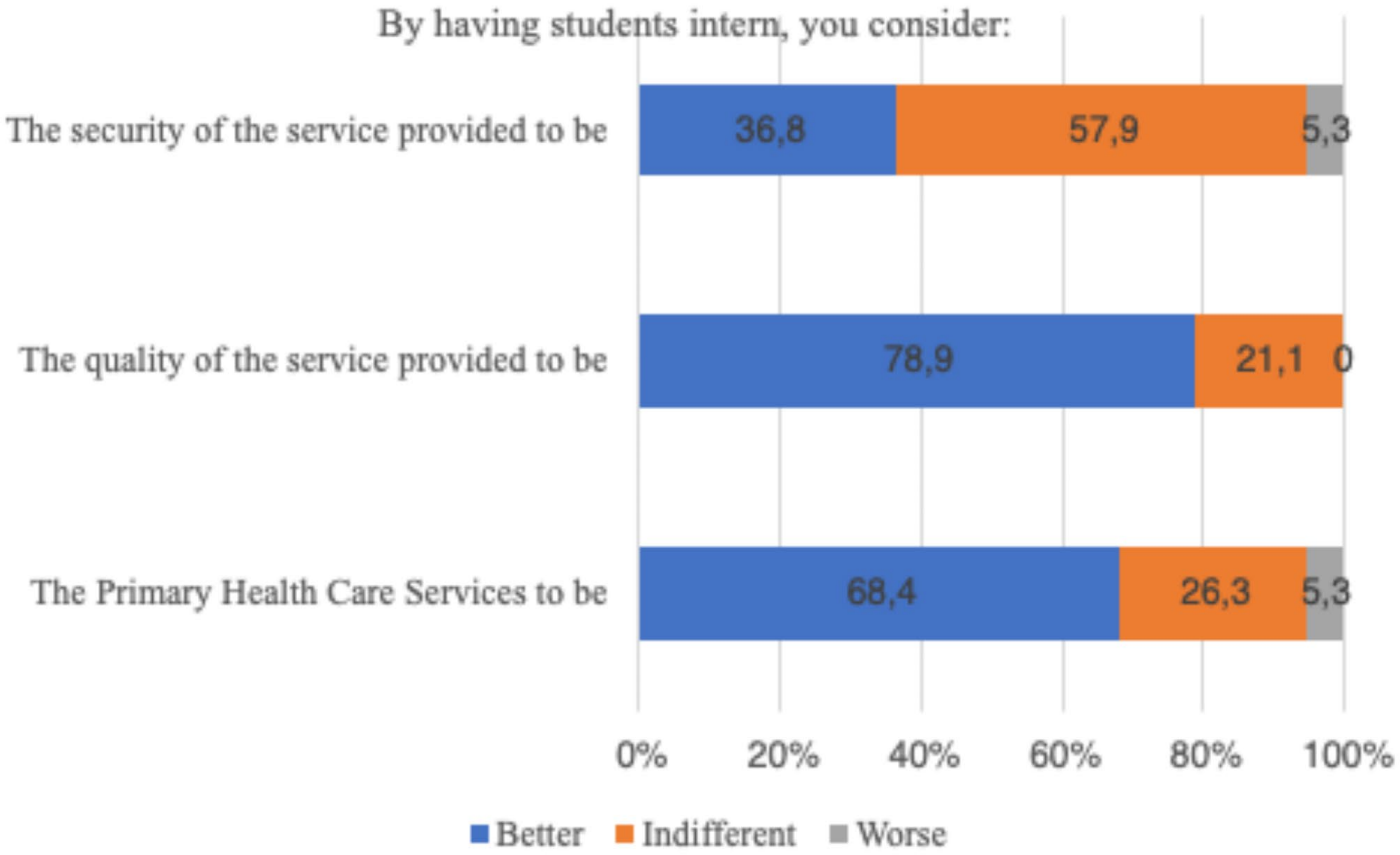
Perception of community residents

A majority of the community (52.6%) were unaware of the internship opportunities at the Primary Health Care Services. Nevertheless, there is a positive perception regarding the quality of services provided, including internships. The presence of students is perceived as advantageous, contributing to more attentive care and enhancing certain services, as illustrated in Graph 2.

#### Passive agent

When inquired about their awareness of the local management council, the responses from the community residents were:

I only saw a poster at the health service. (C4)

I know nothing about this. (C18)

The perception of students and preceptors regarding the community underscores the educational benefits of teaching-service-management-community integration; however, it positions the community solely as a passive agent of observation and a field for experimentation, disconnected from dialogue to ascertain its genuine health needs.

The reality shock was very important to understand the needs of the population that uses the unit. Each home visit showed a different situation, but all made the demands very clear — not just of oral health, but of the families. (S2)

The community will always help […] The community needs to know that it is good for them. (P10)

## Discussion

*This case study elucidates several salient points: the historically colonialist and exclusionary nature of Brazil’s educational system*,* with an emphasis on the predominance of white European male perspectives in public health education; the teaching-service-management-community integration as a crucial factor for transforming health education*,* demonstrating a close connection and effective coordination between the Educational Institutions and SUS health professionals*,* thereby enhancing public health practices; and the limited extent of community involvement in the teaching process.*

### Sociodemographic profile of participants

This profile merits attention as it reflects a historically colonialist power structure: individuals of European descent who introduced a white European male academic elite to Brazil for the purpose of establishing universities, thereby perpetuating patriarchal, racist, and sexist dynamics in Brazilian society over time. The curriculum of graduate courses in Public Health is notably Eurocentric, and there is a pressing need to incorporate authors such as Frantz Fanon, Clovis Moura, Abdias do Nascimento, Lélia Gonzalez, and Beatriz do Nascimento—non-European black intellectuals—into the political discourse surrounding health. The author also emphasizes that, as a general rule, no student completes this course without encountering authors like Marx and Foucault, who are of European origin. This observation motivates an inquiry into the decolonization of public health and, consequently, of health policies and practices, requiring a renegotiation of the democratic nation project as established in the aspirations of the Public Health Reform [20].

A counter-hegemonic agenda is presented through ethnic and student collective bodies, addressing the dynamics of privileges and oppression reproduced by educational institutions. The field of public health is not exempt from this phenomenon. In contemporary society, black, female, Indigenous, non-gendered, and peripheral bodies are marginalized, subordinated, and rendered vulnerable in such ways that health systems and public health practices themselves often contribute to the invisibility of these structures [21].

Identifying, elucidating, and highlighting this issue is essential for political, affective, and cognitive reparation and recovery. Such actions are recognized as part of the decolonization of public and alternative health, aiming to reduce health inequalities [22, 23]. Consequently, representativeness is crucial. Racism, which is a manifestation of an unequal social order, can be addressed—albeit not in isolation—through the representation of black individuals in leadership and decision-making positions [20].

Similar to all other epistemological domains, public health should not be subject to the structuring of gender and ethnic divides. While the topic of racism is critically examined in terms of its causes and consequences, there is a notable absence of organizational affirmative action to promote ethnic representation in the course and its internships. From this reflection, which this research provides, changes can and should be implemented [20].

The absence of black individuals—both male and female—among community residents, in a context predominantly composed of white and brown individuals, was notable. This can be attributed to the broader social support networks of white and brown individuals, which are generally more robust than those available to black individuals, thus limiting their availability. Furthermore, it is imperative to note that women with darker skin face additional discrimination, possess less social support, and encounter greater barriers in accessing rights [24], such as health care, in this specific case. The significance of social support, viewed through a racial lens, is evident in the research findings: 63% of individuals assisted by the students and preceptors were children, and 42.1% of legal guardians had no direct kinship with them, suggesting substantial social support from families.

### Integration from the perspective of the players: teaching-service connection

Teaching-service-management-community integration is internationally recognized as a fundamental pillar in transforming higher education in Dentistry and is becoming increasingly significant in health education in Brazil [11, 25, 26]. Collaboration between Educational Institutions and health services occurs when these institutions allocate resources to train health professionals while actively aiming to transform their practices. In this context, pedagogical support through preceptorship workshops plays a crucial role [27, 28].

The coordination of the specialization course in public health dentistry, in conjunction with its educators, fulfilling their roles as Educational Institutions, facilitated continuing education initiatives for health professionals in Primary Health Care Services. The study also highlighted the learning experiences of preceptors from the perspective of the National Policy for Permanent Health Education [29], which seeks to leverage the daily realities of health services to reorganize work processes [30].

Teaching-service-management-community integration can provide training for students and ongoing education for preceptors. The benefits of preceptorship workshops were particularly notable, as they fostered integration between teaching and service [28]. Enabling preceptors to exercise their roles and integrating them with students’ courses is essential for both the continuing education of professionals and the training of students during their internships. Therefore, the proximity between teaching and service, when preceded by adequate planning and organization, represents an advantageous approach that aligns with the teaching-learning process [4, 28, 31, 32]. Through training, preceptors transitioned from a position of limited knowledge and uncertainty regarding pedagogical roles to an understanding of their responsibilities, accompanied by a greater appreciation of the work involved [32], as evidenced in their testimonials.

Recognizing the role of preceptors is crucial, as they are integral to teaching-service-management-community integration. It is imperative to emphasize their presence in health service-learning environments, providing qualifications through appropriate training and offering commensurate financial compensation for their work [31].

For students, the experience during their internships, shaped by teaching-service-management-community integration, provided profoundly meaningful learning. In this potentially efficacious connection between teaching and service, it is important to note that the supervised curricular internship focuses on developing technical-scientific skills and competencies. Social context experiences stimulate critical thinking, fostering awareness of the significance of protection, prevention, health promotion, and comprehensive, humanized care [4]. Furthermore, these experiences contribute to developing a sense of social and civic responsibility.

As illustrated in Graph 1, it is evident that learning about the territory was considered a crucial training activity by all three segments. Collective action within the territory, home visits, and familiarization with Primary Health Care Services were highly valued. The purpose of these activities is public health training, which involves studying phenomena that affect public health “through analysis, organization, planning, execution, and evaluation of health systems, focused on population groups, with an emphasis on health promotion” [33]. This training explores three key areas within the specialty: Epidemiology; Social and Human Sciences in Health and Policy; and Planning, Management, and Health Assessment.

In public health, understanding the territory—encompassing not only its geographic aspects but also its socioeconomic, cultural, and political dimensions as facets of human activity, which position the territory as a category of social analysis^1^—is fundamental to achieving effective care in Primary Health Care. Consequently, exposing students to the diverse realities of territories served by Primary Health Services was recognized as a vital training strategy.

As noted in the section ‘Focus areas for enhancement’, there are concerns within the Federal Council of Dentistry regarding the insufficient training in dental undergraduate courses. The Council advocates for enhanced professional training in health to strengthen actions aimed at ensuring the quality of professional education. Inadequate undergraduate training poses risks and can lead to irreversible damage to public health [33].

### Integration in Disconnection with management and the community

Despite the efforts made, there was no response from municipal managers. Teaching-service-management-community integration necessitates changes and adaptations from all parties involved, with particular emphasis on the managers, to facilitate their engagement with the training field. Ordinance 1.127/15, which establishes the Organizational Contracts for Public Action in Education and Health, guarantees “access to all health establishments under the responsibility of the manager” and states in Article 13, item VI, that municipal managers have the competence to promote reflection on practice and the exchange of knowledge among health professionals, aiming to identify and discuss work issues to improve health care [34]. The lack of response observed in this research underscores the existing gap in the training field.

Constitutionally, the State’s role in regulating health training processes is crucial for mediating the integration between health services in government entities (municipal, state, and federal), training institutions [35], and society. Moreover, Resolution 569/17 [36] emphasizes the importance of health manager participation in educational institutions to ensure effective integration, which is essential given the challenges present in practice scenarios.

The community aspect is also disconnected from the training process. In this study, the majority (52.6%) of community members were unaware of the internship within the Primary Health Care Services. This finding supports existing literature, which demonstrates a lack of awareness about teaching activities, despite the generally positive perception of the quality of services offered by Primary Health Care Services that provide internships. The presence of students is perceived as beneficial, leading to greater attention to cases and improvements in some services [8–12, 31, 37–40], as shown in Graph 2. Given that popular participation is legally regulated, we inquired about the management council and found a lack of knowledge regarding its role. The management council, a tripartite body composed of managers, service professionals, and equal numbers of users and community members, facilitates popular participation and is recognized by both the Constitution and Law 8142/90 [41].

The lack of knowledge about the internship reinforces that “the training of health professionals has remained separate from the organization of sectoral management and the critical debate on health care structuring systems, proving to be absolutely impervious to social control over the sector, which is the basis of the official Brazilian health model” [2]. Despite the use of teaching-service-management-community integration aimed at training professionals for the health needs of the population, it is important to value them in this process by encouraging dialogue, listening to them, and inviting them to participate. For Strasser et al. (2015) [42] “community” can have multiple interpretations in health education. Some view the community as simply a location where patients reside. Others consider it to encompass everything outside of the healthcare setting. Finally, some perceive community as an essential social construction that merits as much attention as the individuals within it. It is imperative to invite it to participate in pedagogical construction.

In addition to the construction of this training process with the collaboration of the population, Pessoa and Noro (2020) [43] propose a participatory self-assessment process that accompanies the implementation of the actions, with the aim of achieving effectiveness and impact on the training of professionals to address the health needs of the population. This objective will be attainable through collaborative pedagogical planning and development.

### Study limitations

The limitations of this study encompass the non-participation of all teachers and students involved in the development of the course, as well as the absence of the management perspective in the analysis of the training quadrilateral. Regarding qualitative data collection, both the method employed and the circumstances of its application present interconnected challenges. The utilization of a semi-structured online questionnaire may have restricted the depth of the responses, impeding a more detailed exploration of the topic and the capture of the complexity of the participants’ perceptions. Moreover, the environment and timing in which the questionnaires were completed may have influenced the responses, as emotions and personal experiences could have affected the perceptions presented, which can be considered for future research.

### Study recommendations and implications for practice, education, and policy

Based on the findings of this case study, the following recommendations can serve as valuable guidelines for educational institutions and managers seeking to support the teaching-service-management-community integration, with implications for practice, education, and policy:

1. Implement affirmative actions for the selection of black, female, indigenous, non-gendered, and peripheral-bodied teachers, with knowledge of authors who reflect and contribute to their local representation.
2. Facilitate the provision of training for healthcare professionals who will serve as preceptors. It is recommended that this training be offered at a frequency that allows for continuous dialogue between the healthcare service and the educational institution. In this perspective of connection between the stakeholders, it is important to support and financially recognize the professionals who undertake the preceptorship.
3. Fostering the teaching-service-management-community integration is crucial, as the benefits for all stakeholders became evident. However, it is equally important for the educational institution to engage with the local population, addressing their needs, in order to achieve a balance between learning and addressing local health issues. This approach ensures that health education will be meaningful not only for those who utilize the service but also for the community residents, ensuring they are not merely viewed as a pedagogical resource.
4. The study suggests the necessity for a more integrated approach, where the community, managers, health services, and educational institutions collaborate more effectively to address local health needs.

## Conclusion

The integration process necessitates meticulous and thorough analysis for a formative convergence to occur. Constructing social representation from the perspective of the decolonization of public health and anti-racist struggle, in addition to being an alternative to mitigate health inequalities, becomes a significant movement.

The opportunity to engage in on-site internships facilitates the application of theoretical knowledge in a practical context from a formative perspective in and for SUS. It engenders reflections on reality that, from an emancipatory standpoint, foster a level of civilizing consciousness more profound than that attainable within a classroom setting.

In the present study, the teaching-service-management-community integration constitutes the pedagogical potential necessary for the process of continuing education of service professionals and ongoing education in the training of students. This was evidenced by the reflection on the challenges that health workers encounter when they assume a dual role as professionals responsible for public health in the territory and as educators, as well as by the students’ learning reports. Another aspect revealed was the disengagement of sectoral management and the passivity of the community in training and health care, with its impermeability in pedagogical acts within their own territory.

The teaching-service-management-community integration is not integrated with balanced formative convergence forces, relying predominantly on two sides of the quadrilateral: the teaching side, with the willingness to expand learning, and the service side, with its health professionals trained in the art of caring and teaching. The objective is to preserve the aforementioned approach in the future, maintaining adequate prior preparation of preceptors, with greater ethnic and gender representation, while also incorporating the support, interests, and needs of the management and community.

## Electronic supplementary material

Below is the link to the electronic supplementary material.

Supplementary Material 1

Supplementary Material 2

Supplementary Material 3

## Data Availability

No datasets were generated or analysed during the current study.

## Footnotes

1 Mendes, Eugênio Vilaça. A construção social da Atenção Primária à Saúde / Eugênio Vilaça Mendes. Brasília: Conselho Nacional de Secretários de Saúde– CONASS, 2015.
