# Supplementary material for: Connecting knowledge and practice: specialization course in dentistry in public health at Brazilian unified health system - a journey of transformative integration
Source: BMC Med Educ. 2025 Mar 21;25:419. doi: 10.1186/s12909-025-06987-1 (PMC11929345; doi:10.1186/s12909-025-06987-1)
Supplement: Supplementary file 1 — Supplementary Material 1 [file 12909_2025_6987_MOESM1_ESM.pdf]

PARECER CONSUBSTANCIADO DO CEP

DADOS DO PROJETO DE PESQUISA

Título da Pesquisa: COMPREENSÃO DA INTEGRAÇÃO ENSINO-SERVIÇO-GESTÃO-COMUNIDADE ATRAVÉS DE ESTÁGIO NO SUS POR MEIO DE PÓS GRADUAÇÃO EM ODONTOLOGIA NO MUNICÍPIO DE SÃO PAULO/SP

Pesquisador: AFONSO LUIS PUIG PEREIRA

Área Temática:

Versão: 3

CAAE: 52595921.4.0000.0071

Instituição Proponente: SOCIEDADE BENEF ISRAELITABRAS HOSPITAL ALBERT EINSTEIN

Patrocinador Principal: Financiamento Próprio

DADOS DO PARECER

Número do Parecer: 5.288.100

Apresentação do Projeto:

As informações elencadas nos campos "Apresentação do Projeto", "Objetivo da Pesquisa" e "Avaliação dos Riscos e Benefícios" foram retiradas do arquivo Informações Básicas da Pesquisa (PB_INFORMAÇÕES_BÁSICAS_DO_PROJETO_1827793.pdf de 04/03/2022) e/ou do Projeto Detalhado/ Brochura do Investigador (Projeto_completo_versao_limpa.docx de 17/02/2022).

Resumo:

Objetivo: Este projeto propõe conhecer a integração ensino-serviço-gestão-comunidade através da percepção dos atores envolvidos na pós graduação de odontologia de um curso de especialização. Método: O curso de especialização de Odontologia em Saúde Coletiva do Instituto de Ensino e Pesquisa Albert Einstein tem previsão de estágio nas Unidades Básicas de Saúde (UBS) em 2021 e 2022. Assim, os estudantes serão inseridos no serviço e acolhidos por preceptores selecionados previamente e preparados pedagogicamente para o exercício desta função. Os participantes do estudo serão usuários do SUS, estudantes, docentes, preceptores e gestores de saúde. Haverá, após o final do estágio, através de formulário e aplicado questionário semiestruturado e, se necessário, um grupo focal on-line. O Termo de Consentimento Livre e Esclarecido (TCLE) sempre deverá ser preenchido e assinado previamente à coleta de dados.

Portanto, é um estudo com desenho qualitativo com análise estatística descritiva e análise de conteúdo temática. Conclusão: Espera-se conhecer a percepção de usuários, discentes, docentes e preceptores sobre o processo ensino-aprendizagem e analisar a integração ensino-serviço-comunidade identificando pontos de convergência e divergência formativa.

Hipótese:

Muita convergência formativa entre ensino-serviço e pouca convergência formativa dos atores envolvidos no ensino-serviço com a gestão e usuários do SUS.

Metodologia Proposta:

Método Tipo de estudo Trata-se de um estudo descritivo, exploratório, de abordagem qualitativa. Referencial teórico-metodológico A fundamentação teórica tem por base três embasamentos principais: quadrilátero da Formação (Ceccim; Fewerwerker, 2004), andragogia (FREIRE, 2016) e Educação Permanente em Saúde (EPS) (BRASIL, 2009). Cenário do estudo Este trabalho parte de uma atividade pedagógica do curso de especialização de Odontologia em Saúde Coletiva: ênfase em saúde da família e comunidade, do Institutito Israelita de Ensino e Pesquisa, que é realizada nos serviços de saúde do Sistema Único de Saúde (SUS), localizados na região sul do município de São Paulo na área de abrangência da parceria público-privada entre Secretaria Municipal de Saúde do município de São Paulo (SMSSP) e a Sociedade Beneficente Israelita Brasileira Albert Einstein (SBIBAE). O curso de Odontologia em Saúde Coletiva: ênfase em saúde da família e comunidade tem duração de 12 meses e é oferecido à cirurgiõesdentistas que possuem Registro Definitivo no Conselho Regional da Classe. O curso está organizado em encontros teóricos com periodicidade mensal, que se intercalam entre momentos presenciais e remotos. Além disso, conta com um diferencial em sua grade curricular que refere-se à oferta de estágio em unidades básicas de saúde, com carga horária de 40h. O estágio está programado para acontecer em quatro momentos distintos ao longo do curso e o estudante poderá optar pelo período que irá realizar o estágio. Os grupos tutoriais serão constituídos por três a cinco estudantes por preceptor. Destaca-se que cada UBS contará com um ou dois preceptores, previamente treinados, para recepção dos estudantes. As atividades de imersão em campo serão realizadas nas seguintes unidades básicas de saúde: Paraisópolis 3, Campo Limpo e Jardim Olinda. Nesses serviços, os estudantes, acompanhados dos preceptores, farão atendimento clínico, reconhecimento de território, reconhecimento da UBS,

reunião de equipe, acompanhamento da gestão, acolhimento de demanda espontânea, acompanhamento de consulta de enfermagem, de medicina e de odontologia, visita ao Centro de Atenção Psicossocial (CAPS), visita domiciliar, Atraumatic Restauration Treatment (ART), grupo educativo, triagem e outras atividades que forem pertinentes à educação.

Critério de Inclusão:

A população do estudo será constituída por docentes, preceptores (profissionais de saúde) e estudantes matriculados no referido curso além dos gestores pertencentes à área técnica da instituição (como Coordenadores de UBS do Instituto de Responsabilidade Social - IIRS), da Supervisão Técnica de Saúde Campo Limpo (STSCL) e Coordenadoria Regional de Saúde Sul (CRSSul) e usuários cadastrados nas UBS que serão realizadas as atividades de imersão. Será utilizada amostragem por conveniência. A abordagem dos profissionais de saúde (preceptores) será realizada, por pesquisador voluntário, em reunião de alinhamento posterior ao estágio, onde serão convidados a participar da pesquisa. O contato será realizado com o mesmo rigor em relação à apresentação dos objetivos do estudo, procedimentos de coleta de dados, aspectos éticos envolvidos e formalização do convite de participação no estudo. Para a abordagem dos gestores dos serviços, sendo eles pertencentes ao IIRS, STSCL ou CRSSul, os pesquisadores realizarão o contato prévio, in loco, para agendamento de um encontro com o objetivo de apresentar a proposta do presente estudo, esclarecer dúvidas e a formalizar o convite para participar do estudo. Por fim, a abordagem dos usuários ocorrerá durante os períodos de estágio nas UBS. Será realizado um planejamento visando organizar o período de coleta de dados em cada unidade, garantindo a presenta de um pesquisador responsável pela abordagem e convite ao usuário. O contato direto feito por um pesquisador voluntário aos usuários será realizado após o atendimento do mesmo em ações de saúde bucal. Será apresentada a proposta e objetivos estudo, procedimentos de coleta de dados, aspectos éticos e a formalização do convite para participação no estudo.

Critério de Exclusão:

Usuários que não pertencem a uma das UBS em que ocorrer o estágio. Estudantes, preceptores, profissionais de saúde das UBS envolvidas, gestores e docentes que se recusarem a participar do estudo.

Objetivo da Pesquisa:

Objetivo Primário:

Conhecer e compreender a integração ensino-serviço-gestão-comunidade por meio da percepção dos atores envolvidos no de um curso de especialização de Odontologia em Saúde Coletiva: ênfase em saúde de família e comunidade no município de São Paulo/SP.

Objetivo Secundário:

a. Conhecer e compreender a percepção da integração ensino-serviço-gestão-comunidade na perspectiva de preceptores do curso. b. Conhecer a percepção sobre educação permanente para os preceptores. c. Conhecer e compreender a integração ensino-serviço-gestão-comunidade na perspectiva de docentes do curso. d. Conhecer e compreender a integração ensino-serviço-gestão-comunidade na perspectiva da comunidade. e. Conhecer e compreender a integração ensino-serviço-gestão-comunidade na perspectiva dos estudantes do curso. f. Conhecer a contribuição do estágio na perspectiva de estudantes. g. Conhecer e compreender a integração ensino-serviço-gestão-comunidade na perspectiva de gestores.

Avaliação dos Riscos e Benefícios:

Riscos:

Todos os cuidados necessários serão tomados, entretanto, existem riscos mínimos característicos do ambiente virtual em função das limitações das tecnologias utilizadas, como a perda de confidencialidade. Além disso, possível desconforto pode ocorrer por conta do tempo para responder as perguntas ou constrangimento em expor suas ideias e percepções, no entanto, você pode deixar de responder as questões que não se sentir à vontade.

Benefícios:

Acredita-se que este estudo ampliará o campo de conhecimentos do ensino e contribuirá para o aprimoramento da formação discente e docente.

Endereço: Av. Albert Einstein 627 - 2ss
Bairro: Morumbi **CEP:** 05.652-000
UF: SP **Município:** SAO PAULO
Telefone: (11)2151-3729 **Fax:** (11)2151-0273 **E-mail:** cep@einstein.br

Comentários e Considerações sobre a Pesquisa:

Desenho:

Pesquisa descritiva e exploratória de abordagem qualitativa com utilização de questionário on-line semiestruturado com perguntas abertas e fechadas para preceptores, gestores, docentes, estudantes e usuários do SUS a partir de 16 anos. Também serão analisados diários de campo escrito por estudantes e preceptores. A orientação metodológica se dará através de análise de conteúdo, se efetivando a partir da gravação e transcrição das falas obtidas em entrevistas ou grupo focal, leitura exaustiva, exploração do material e síntese interpretativa (Minayo et al., 2016). Os resultados qualitativos serão parametrizados através do o guia Consolidated criteria for reporting qualitative research (COREQ) (Souza et al., 2021)

Metodologia de Análise de Dados:

A orientação metodológica se dará através de análise de conteúdo, se efetivando a partir da gravação e transcrição das falas obtidas em entrevistas ou grupo focal, leitura exaustiva, exploração do material e síntese interpretativa (Minayo et al., 2016).

Desfecho Primário:

Percepção de estudantes, preceptores, docentes e gestores e usuários do SUS

Tamanho da Amostra no Brasil: 50

Considerações sobre os Termos de apresentação obrigatória:

vide: Conclusões ou Pendências e Lista de Inadequações.

Recomendações:

vide: Conclusões ou Pendências e Lista de Inadequações.

Conclusões ou Pendências e Lista de Inadequações:

Resposta ao parecer 5.230.694 de 08 de Fevereiro de 2022

Endereço: Av. Albert Einstein 627 - 2ss
Bairro: Morumbi **CEP:** 05.652-000
UF: SP **Município:** SAO PAULO
Telefone: (11)2151-3729 **Fax:** (11)2151-0273 **E-mail:** cep@einstein.br

1. Segundo Resolução CNS nº 580 de 2018, Art. 4º, o TCLE precisa informar ao participante da pesquisa recrutado em serviço de saúde vinculado ao SUS (usuário do serviço de saúde) a diferença entre o procedimento da pesquisa e o atendimento de rotina do serviço. Solicita-se adequação. Embora o procedimento realizado na pesquisa seja o preenchimento de um questionário, deverá ser esclarecido ao participante se existe diferença no atendimento de rotina do serviço em relação à participação no estudo. E no caso do participante se recusar a participar da pesquisa, a garantia de que nada será alterado no seu atendimento.

Resposta: descata-se no TCLE Usuários, na página 21, o seguinte trecho: “Reforçamos que as ações descritas acima fazem parte do protocolo deste estudo. Isto é, o preenchimento do questionário se caracteriza como uma etapa desta pesquisa e está sob a responsabilidade da equipe de pesquisadores. Ela não faz parte do atendimento oferecido pela unidade de saúde e, portanto, você poderá deixar de participar dessa atividade em qualquer momento, sem prejuízos, penalizações ou interrupções em seu tratamento de saúde.”

ANÁLISE: PENDÊNCIA ATENDIDA

2. Segundo Resolução CNS nº 510 de 2016, Art. 5º, O processo de comunicação do consentimento e do assentimento deve estar claro no projeto. Solicita-se adequação. Não está claro quem é o “pesquisador voluntário” informado no corpo do projeto, se será designado um membro da equipe da pesquisa, ou se a abordagem aos participantes da pesquisa será feita aleatoriamente por qualquer profissional que não faz parte da equipe

Resposta: na página 8, foi incluído o seguinte trecho para esclarecimento deste pesquisador: “, membro da equipe da pesquisa que não esteja em atividade direta com os grupos envolvidos na coleta de dados”. Na página 9 foi acrescentado: “membro da equipe da pesquisa sem relação profissional direta com os preceptores”

ANÁLISE: PENDÊNCIA ATENDIDA

3. Segundo Resolução CNS nº 466 de 2012, itens III.2.i e IV.3.e, é necessário esclarecer quais os procedimentos serão adotados para a garantia da confidencialidade, privacidade e segurança no tratamento dos dados.

Resposta: em atendimento a esta solicitação foi reformulado os seguintes trechos:

Página 20: “Ao finalizar seu atendimento com o profissional de saúde bucal, você será abordado por um pesquisador que apresentará a proposta do estudo e formalizará o convite de participação nessa pesquisa. Destaca-se que, se você não tiver interesse em participar, poderá sinalizar ao

pesquisador sem nenhuma consequência, prejuízo ou modificação em seu acompanhamento neste serviço de saúde. Será realizada a leitura desse documento e, caso você concorde em participar, uma via impressa do termo de consentimento livre e esclarecido será entregue a você."
Página 21: "é garantido que você não será penalizado ou sofrerá qualquer prejuízo em seus atendimentos neste serviço de saúde."
Página 22: "privacidade e segurança. Ressalta-se que o gerenciamento das informações seguirá as determinações da Lei Geral de Proteção de Dados (Lei 13.709/18) e as orientações oportunas para procedimentos de pesquisa com alguma etapa em ambiente virtual"
ANÁLISE: PENDÊNCIA ATENDIDA

Após análise, não foram observados óbices éticos.

Considerações Finais a critério do CEP:

Diante do exposto, o Comitê de Ética em Pesquisa do Hospital Israelita Albert Einstein, de acordo com a Resolução CNS nº 466 de 2012 e Norma Operacional nº 001 de 2013 do CNS, manifesta-se pela aprovação do projeto de pesquisa proposto.

Este parecer foi elaborado baseado nos documentos abaixo relacionados:

Tipo Documento	Arquivo	Postagem	Autor	Situação
Informações Básicas do Projeto	PB_INFORMAÇÕES_BÁSICAS_DO_PROJETO_1827793.pdf	04/03/2022 15:34:55		Aceito
Outros	Carta_resposta_CEP_Einstein.docx	04/03/2022 15:34:22	AFONSO LUIS PUIG PEREIRA	Aceito
Outros	TCLE_Usuario_destacada.docx	17/02/2022 11:17:39	AFONSO LUIS PUIG PEREIRA	Aceito
TCLE / Termos de Assentimento / Justificativa de Ausência	TCLE_DPEG.docx	17/02/2022 11:17:29	AFONSO LUIS PUIG PEREIRA	Aceito
TCLE / Termos de Assentimento / Justificativa de Ausência	TCLE_Usuario_limpa.docx	17/02/2022 11:17:21	AFONSO LUIS PUIG PEREIRA	Aceito
Outros	Projeto_completo_versao_destacada.docx	17/02/2022 11:17:06	AFONSO LUIS PUIG PEREIRA	Aceito
Projeto Detalhado / Brochura Investigador	Projeto_completo_versao_limpa.docx	17/02/2022 11:16:45	AFONSO LUIS PUIG PEREIRA	Aceito

Declaração de Pesquisadores	Termo_respons_Pesq.pdf	07/10/2021 15:08:13	AFONSO LUIS PUIG PEREIRA	Aceito
Folha de Rosto	folha_de_rosto_ass_07_10.pdf	07/10/2021 14:01:40	AFONSO LUIS PUIG PEREIRA	Aceito
Outros	Termo_anuencia_gestores.pdf	05/10/2021 18:54:04	AFONSO LUIS PUIG PEREIRA	Aceito
Declaração de Pesquisadores	Termo_compromisso_pesq_resp.pdf	05/10/2021 18:53:49	AFONSO LUIS PUIG PEREIRA	Aceito
Declaração de Instituição e Infraestrutura	Autorizacao_CRS_Sul.pdf	20/09/2021 12:52:56	AFONSO LUIS PUIG PEREIRA	Aceito

Situação do Parecer:
Aprovado

Necessita Apreciação da CONEP:
Não

SAO PAULO, 12 de Março de 2022

Assinado por:
Fabio Pires de Souza Santos
(Coordenador(a))

Endereço: Av. Albert Einstein 627 - 2ss
Bairro: Morumbi **CEP:** 05.652-000
UF: SP **Município:** SAO PAULO
Telefone: (11)2151-3729 **Fax:** (11)2151-0273 **E-mail:** cep@einstein.br
