# Supplementary material for: Connecting knowledge and practice: specialization course in dentistry in public health at Brazilian unified health system - a journey of transformative integration
Source: BMC Med Educ. 2025 Mar 21;25:419. doi: 10.1186/s12909-025-06987-1 (PMC11929345; doi:10.1186/s12909-025-06987-1)
Supplement: Supplementary file 2 — Supplementary Material 2 [file 12909_2025_6987_MOESM2_ESM.pdf]

Machine Translated by Google

CONSUBSTANTIATED OPINION OF THE CEP

RESEARCH PROJECT DATA

Search Title: UNDERSTANDING TEACHING-SERVICE-MANAGEMENT-COMMUNITY INTEGRATION THROUGH AN INTERNSHIP AT SUS THROUGH POSTGRADUATE IN DENTISTRY IN THE MUNICIPALITY OF SÃO PAULO/SP

Researcher: AFONSO LUIS PUIG PEREIRA

Thematic Area:

Version: 3

CAAE: 52595921.4.0000.0071

Proposing Institution: SOCIETY BENEF ISRAELITABRAS HOSPITAL ALBERT EINSTEIN

Main Sponsor: Own Financing

OPINION DATA

Opinion Number: 5,288,100

Project Presentation: The

information listed in the fields "Project Presentation", "Research Objective" and "Evaluation of Risks and Benefits" were taken from the Basic Research Information file (PB_INFORMAÇÕES_BÁSICAS_DO_PROJETO_1827793.pdf dated 03/04/2022) and/or of the Detailed Project/Researcher's Brochure (Projeto_completo_versao_limpa.docx dated 02/17/2022).

Summary:

Objective: This project proposes to understand the teaching-service-management-community integration through the perception of the actors involved in the postgraduate dentistry course of a specialization course. Method: The specialization course in Dentistry in Public Health at the Albert Einstein Teaching and Research Institute is scheduled for an internship in Basic Health Units (UBS) in 2021 and 2022. Thus, students will be inserted into the service and welcomed by previously selected preceptors and pedagogically prepared to perform this role. Study participants will be SUS users, students, teachers, preceptors and health managers. After the end of the internship, there will be a form and a semi-structured questionnaire and, if necessary, an online focus group. The Free and Informed Consent Form (TCLE) must always be completed and signed prior to collection

of data.

Machine Translated by Google

Therefore, it is a study with a qualitative design with descriptive statistical analysis and thematic content analysis. Conclusion: It is expected to understand the perception of users, students, teachers and preceptors about the teaching-learning process and analyze the teaching-service-community integration, identifying points of convergence and divergence in training.

Hypothesis:

A lot of training convergence between teaching-service and little training convergence of the actors involved in teaching-service with the management and users of the SUS.

Proposed Methodology:

Method Type of study This is a descriptive, exploratory study with a qualitative approach. Theoretical-methodological framework The theoretical foundation is based on three main foundations: Training quadrangle (Ceccim; Fewerwerker, 2004), andragogy (FREIRE, 2016) and Permanent Health Education (EPS) (BRASIL, 2009). Study scenario This work is part of a pedagogical activity of the specialization course in Dentistry in Public Health: emphasis on family and community health, from the Instituto Israelita de Ensino e Pesquisa, which is carried out in the health services of the Unified Health System (SUS).), located in the southern region of the city of São Paulo in the area covered by the public-private partnership between the Municipal Health Department of the city of São Paulo (SMSSP) and the Sociedade Beneficente Israelita Brasileira Albert Einstein (SBIBAE). The Public Health Dentistry course: emphasis on family and community health lasts 12 months and is offered to dental surgeons who have Final Registration with the Regional Class Council. The course is organized into monthly theoretical meetings, which alternate between face-to-face and remote sessions. Furthermore, it has a difference in its curriculum that refers to the offer of internships in basic health units, with a workload of 40 hours. The internship is scheduled to take place at four different times throughout the course and the student can choose the period during which the internship will take place. Tutorial groups will consist of three to five students per preceptor. It is noteworthy that each UBS will have one or two previously trained preceptors to receive students. Field immersion activities will be carried out in the following basic health units: Paraisópolis 3, Campo Limpo and Jardim Olinda.

In these services, students, accompanied by preceptors, will undergo clinical care, territory recognition, UBS recognition,

Machine Translated by Google

team meeting, management monitoring, acceptance of spontaneous demand, monitoring of nursing, medical and dental consultations, visit to the Psychosocial Care Center (CAPS), home visit, Atraumatic Restoration Treatment (ART), educational group, screening and other activities that are relevant to education.

Inclusion Criteria:

The study population will consist of teachers, preceptors (health professionals) and students enrolled in the aforementioned course, in addition to managers belonging to the technical area of the institution (such as UBS Coordinators of the Institute of Social Responsibility - IIRS), Technical Supervision of Field Health Limpo (STSCL) and South Regional Health Coordination (CRSSul) and users registered at the UBS where the immersion activities will be carried out. Convenience sampling will be used. The approach of health professionals (preceptors) will be carried out, by a volunteer researcher, in an alignment meeting after the internship, where they will be invited to participate in the research. Contact will be carried out with the same rigor in relation to the presentation of the study objectives, data collection procedures, ethical aspects involved and formalization of the invitation to participate in the study. To approach service managers, whether they belong to IIRS, STSCL or CRSSul, the researchers will make prior contact, on site, to schedule a meeting with the aim of presenting the proposal for this study, clarifying doubts and formalizing the invitation to participate in the study. Finally, users will be approached during internship periods at the UBS. Planning will be carried out to organize the data collection period in each unit, ensuring the presence of a researcher responsible for the approach and invitation to the user. Direct contact made by a volunteer researcher to users will be carried out after providing oral health care. The study proposal and objectives, data collection procedures, ethical aspects and the formalization of the invitation to participate in the study will be presented.

Exclusion Criteria:

Users who do not belong to one of the UBS where the internship takes place. Students, preceptors, health professionals from the UBS involved, managers and teachers who refuse to participate in the study.

Address: Av. Albert Einstein 627 - 2ss
Neighborhood: Morumbi **ZIP CODE:** 05.652-000
UF: SP **Municipality:** SAO PAULO
Telephone: (11)2151-3729 **Fax:** (11)2151-0273 **Email:** cep@einstein.br

Machine Translated by Google

Research Objective:

Primary Objective:

Know and understand the teaching-service-management-community integration through the perception of the actors involved in a specialization course in Dentistry in Public Health: emphasis on family and community health in the city of São Paulo/SP.

Secondary Objective:

The. Know and understand the perception of teaching-service-management-community integration from the perspective of course preceptors. B. Know the perception of continuing education for preceptors. w.
Know and understand teaching-service-management-community integration from the perspective of course teachers.
d. Know and understand teaching-service-management-community integration from the community perspective. It is.
Know and understand teaching-service-management-community integration from the perspective of course students. f.
Know the contribution of the internship from the perspective of students. g. Know and understand teaching-service-management-community integration from the perspective of managers.

Assessment of Risks and Benefits:

Scratchs:

All necessary care will be taken, however, there are minimal risks characteristic of
virtual environment due to the limitations of the technologies used, such as loss of confidentiality.
In addition, possible discomfort may occur due to the time it takes to answer questions or embarrassment in expressing your ideas and perceptions, however, you can stop answering questions if you do not feel comfortable.

Benefits:

It is believed that this study will expand the field of teaching knowledge and contribute to the improvement of student and teacher training.

Machine Translated by Google

Comments and Considerations about the Research:

Design:

Descriptive and exploratory research with a qualitative approach using a semi-structured online questionnaire with open and closed questions for preceptors, managers, teachers, students and SUS users aged 16 and over. Field diaries written by students and preceptors will also be analyzed. The methodological guidance will be provided through content analysis, based on the recording and transcription of statements obtained in interviews or focus groups, exhaustive reading, exploration of the material and interpretative synthesis (Minayo et al., 2016). The qualitative results will be parameterized using the Consolidated criteria for reporting qualitative research (COREQ) guide (Souza et al., 2021)

Data Analysis Methodology:

The methodological guidance will be provided through content analysis, based on the recording and transcription of statements obtained in interviews or focus groups, exhaustive reading, exploration of the material and interpretative synthesis (Minayo et al., 2016).

Primary Outcome:

Perception of students, preceptors, teachers and managers and users of the SUS

Sample Size in Brazil: 50

Considerations regarding the Mandatory Presentation Terms: see: Conclusions or Pending Issues and List of Inadequacies.

Recommendations:

see: Conclusions or pending issues and List of Inadequacies.

Conclusions or pending issues and list of inadequacies:

Response to opinion 5,230,694 of February 8, 2022

Machine Translated by Google

1. According to CNS Resolution No. 580 of 2018, Art. 4, the TCLE needs to inform the research participant recruited in a health service linked to the SUS (user of the health service) the difference between the research procedure and the routine care of the service. Adaptation is requested. Although the procedure carried out in the research is filling out a questionnaire, it must be clarified to the participant whether there is a difference in the service's routine care in relation to participation in the study. And in case the participant refuses to participate in the research, the guarantee that nothing will be changed in their
service.

Answer: the following excerpt is disregarded in the Users TCLE, on page 21: "We emphasize that the actions described above are part of the protocol of this study. That is, completing the questionnaire is characterized as a stage of this research and is the responsibility of the research team. It is not part of the care offered by the health unit and, therefore, you may stop participating in this activity at any time, without losses, penalties or interruptions in your treatment.
health."

ANALYSIS: PENDENCY ATTENDED

2. According to CNS Resolution No. 510 of 2016, Art. 5, The process for communicating consent and assent must be clear in the project. Adaptation is requested. It is not clear who the "volunteer researcher" informed in the body of the project is, whether a member of the research team will be designated, or whether the research participants will be approached randomly by any professional who is not part of the team Answer: on the page 8, the following excerpt was
included for clarification by this researcher: ", member of the research team who is not in direct activity with the groups involved in data collection". On page 9 it was added: "member of the research team with no direct professional relationship with the preceptors"

ANALYSIS: PENDENCY ATTENDED

3. According to CNS Resolution No. 466 of 2012, items III.2.i and IV.3.e, it is necessary to clarify which procedures will be adopted to guarantee confidentiality, privacy and security in the treatment
of the data.

Answer: in response to this request, the following excerpts have been reformulated:
Page 20: "When you have completed your consultation with the oral health professional, you will be approached by a researcher who will present the study proposal and formalize the invitation to participate in this research. Please note that if you are not interested in participating, you can signal the

Machine Translated by Google

researcher without any consequences, losses or changes in their follow-up in this health service. This document will be read and, if you agree to participate, a printed copy of the informed consent form will be given to you.”

Page 21: “it is guaranteed that you will not be penalized or suffer any harm in your care at this health service.”

Page 22: “privacy and security. It should be noted that information management will follow the provisions of the General Data Protection Law (Law 13,709/18) and the appropriate guidelines for research procedures with some stage in a virtual environment”
ANALYSIS: PENDENCY ATTENDED

After analysis, no ethical obstacles were observed.

Final Considerations at the discretion of the CEP: In view of the above, the Research Ethics Committee of the Hospital Israelita Albert Einstein, in accordance with CNS Resolution nº 466 of 2012 and Operational Standard nº 001 of 2013 of the CNS, expresses its approval of the project proposed research.

This opinion was prepared based on the documents listed below:

Document Type File PB_INFORMAÇÕES_BÁSICAS_DO_P Basic		Post	Author	Situation
Carta_resposta_CEP_Einstein.docx	Information of the Project ROJETO_1827793.pdf Others	04/03/2022 15:34:55		Accepted
		04/03/2022 15:34:22	AFONSO LUIS PUIG PEREIRA	Accepted
Others	TCLE_Usuario_detached.docx	17/02/2022 11:17:39	AFONSO LUIS PUIG PEREIRA	Accepted
TCLE / Terms of Assent / Justification of Absence	TCLE_DPEG.docx	17/02/2022 11:17:29	AFONSO LUIS PUIG PEREIRA	Accepted
TCLE / Terms of Assent / Justification of Absence	TCLE_Usuario_limpa.docx	02/17/2022 11:17:21	AFONSO LUIS PUIG PEREIRA	Accepted
Others	Project_complete_version_detached.do cx	02/17/2022 11:17:06	AFONSO LUIS PUIG PEREIRA	Accepted
Detailed project / Brochure Investigator	Project_complete_version_clean.docx	02/17/2022 11:16:45	AFONSO LUIS PUIG PEREIRA	Accepted

Machine Translated by Google

Declaration of Researchers	Termo_respons_Pesq.pdf	07/10/2021 15:08:13	AFONSO LUIS PUIG PEREIRA	Accepted
Title Page	foil_de_rosto_ass_07_10.pdf	07/10/2021 14:01:40	AFONSO LUIS PUIG PEREIRA	Accepted
Others	Termo_anuencia_gestores.pdf	05/10/2021 18:54:04	AFONSO LUIS PUIG PEREIRA	Accepted
Declaration of Researchers	Termo_compromisso_pesq_resp.pdf	05/10/2021 18:53:49	AFONSO LUIS PUIG PEREIRA	Accepted
Declaration of Institution and Infrastructure	Authorization_CRS_Sul.pdf	20/09/2021 12:52:56	AFONSO LUIS PUIG PEREIRA	Accepted

Status of the Opinion:
Approved

Requires CONEP Appraisal:
No

SAO PAULO, March 12, 2022

Signed by:
Fabio Pires de Souza Santos
(Coordinator)
