# Supplementary material for: Connecting knowledge and practice: specialization course in dentistry in public health at Brazilian unified health system - a journey of transformative integration
Source: BMC Med Educ. 2025 Mar 21;25:419. doi: 10.1186/s12909-025-06987-1 (PMC11929345; doi:10.1186/s12909-025-06987-1)
Supplement: Supplementary file 3 — Supplementary Material 3 [file 12909_2025_6987_MOESM3_ESM.pdf]

ANNEX 1 - Users/patients

GROUP 1 – Sociodemographic characteristics

Team, Family and Micro _______________

2. Gender

( ) Masculine

( ) Feminine

( ) Prefer not to say ______________

3. Age _________________

4. Type

( ) User

( ) Legal responsible

5. Color/Race

( ) White

( ) Black

( ) Brown

( ) Yellow

( ) Indigenous

GROUP 2 – PERCEPTION OF THE TEACHING-LEARNING PROCESS

( ) Dental consultation ( ) VD ( ) Collective action

6. You were assisted by:

( ) Preceptor / Health professional

( ) Student

( ) For both

( ) I don't know

7. Did you know that the teaching process takes place at this UBS?

( ) Yes

( ) No

8. If so, what did you notice that was different about UBS? _______________ (free text)

9. Question in case of action in the territory: Did you realize that you just went through a consultation / action with students?

( ) Yes

( ) No

10. Question in case of action in the territory: If yes, what have you noticed? _______________ (free text)

11. The fact that this consultation is a teaching process, in general the consultation:

( ) Was better

( ) It was the same

( ) It was worse

( ) Indifferent

12. The fact that this consultation/action was a teaching process, the quality of the consultation:

( ) Was better

( ) It was the same

( ) It was worse

( ) Indifferent

13. The fact that this consultation was a teaching process, did you feel:

( ) Safer

( ) Normal

( ) Indifferent

( ) Insecure

( ) Very insecure

14. Did you consider the consultation/action time?

( ) More than enough

( ) Enough

( ) Indifferent

( ) Insufficient

( ) Very insufficient

15. Have you ever been invited to participate in any council at UBS to decide on community health issues?

( ) Yes

( ) No

16. Have you ever heard of the UBS Management Board?

( ) Yes

( ) No

17. Would you like to say something more about the consultation/action, about UBS or about the teaching process that takes place here? ________________________(free text)

ANNEX 2 - Teachers/Preceptors/Students/Managers

PREVIOUS QUESTIONS TO STUDENTS

What do you hope to learn from this internship experience? __________________(free text)

What are the main aspects of oral health care that you imagine you will find in the UBS you will visit? ________________________(free text)

GROUP 1 – GENERAL CHARACTERISTICS OF THE POPULATION

1. Gender

( ) Masculine

( ) Feminine

( ) Prefer not to say

2. Age ____________

3. Group to which you belong

( ) Teacher

( )	Student

( )	Preceptor

( )	Manager

4. Color/Race

( )	White

( )	Black

( )	Brown

( )	Yellow

( )	Indigenous

GROUP 2 – PERCEPTION OF THE TEACHING-LEARNING PROCESS

5. Describe, in general, your perception of the Public Health Dentistry course: emphasis on family and community health. _________________________(free text)

6. Describe, in detail, your perception of the internship. _________________________(free text)

7. Describe the points of improvement and learning that you noticed in yourself throughout the internship (what you learned and what needs to improve that you experienced during the internship). _________________________(free text)

8. Did you notice any difference in your learning throughout the internship? If so, can you describe what they were? _________________________(free text)

9. Describe your perception of teaching-service-management-community integration when participating in this internship? _________________________(free text)

10. Do you want to make any further comments? This is a space to express yourself freely and leave suggestions, criticisms and praise for the process you experienced during the internship. _________________________(free text)

11. What were the main aspects of oral health care that you found at the UBS you visited? _________________________(free text)

12. List the three activities you enjoyed most and justify at least one of them. _________________________(free text)

13. List the three activities you enjoyed the least. Justify at least one of them. _________________________(free text)

14. What, in the oral health system you have seen, should be changed in your opinion? Justify. _________________________(free text)

16. On a scale from 1 (little importance) to 5 (very important), what rating do you give to one of the following items, in relation to the importance of learning (Lickert scale)

Territory recognition

UBS recognition

Staff meeting

Management monitoring

Reception

Nursing consultation

Doctor's consultation

Dentist consultation

Visit to CAPS

VD

ART

Educational group

Screening

17. Do you want to make any further comments? This is a space to express yourself freely about the course or your experience. ________________________(free text)
